# Flexible selection of a single treatment incorporating short‐term endpoint information in a phase II/III clinical trial

**DOI:** 10.1002/sim.6567

**Published:** 2015-06-26

**Authors:** Nigel Stallard, Cornelia Ursula Kunz, Susan Todd, Nicholas Parsons, Tim Friede

**Affiliations:** ^1^ Statistics and Epidemiology, Division of Health SciencesWarwick Medical School, University of WarwickCoventryU.K.; ^2^ Department of Mathematics and StatisticsUniversity of ReadingReadingU.K.; ^3^ Department of Medical StatisticsUniversity Medical CenterGöttingenGermany

**Keywords:** adaptive design, conditional error, error rate control, multiple testing, sequential clinical trial

## Abstract

Seamless phase II/III clinical trials in which an experimental treatment is selected at an interim analysis have been the focus of much recent research interest. Many of the methods proposed are based on the group sequential approach. This paper considers designs of this type in which the treatment selection can be based on short‐term endpoint information for more patients than have primary endpoint data available. We show that in such a case, the familywise type I error rate may be inflated if previously proposed group sequential methods are used and the treatment selection rule is not specified in advance. A method is proposed to avoid this inflation by considering the treatment selection that maximises the conditional error given the data available at the interim analysis. A simulation study is reported that illustrates the type I error rate inflation and compares the power of the new approach with two other methods: a combination testing approach and a group sequential method that does not use the short‐term endpoint data, both of which also strongly control the type I error rate. The new method is also illustrated through application to a study in Alzheimer's disease. © 2015 The Authors. Statistics in Medicine Published by John Wiley & Sons Ltd.

## 1 Introduction

Adaptive and sequential methods are often used in clinical trials to allow changes to be made to a trial design at one or more interim analyses during the course of the trial on the basis of the data observed. Such methods are appealing because they allow data observed early in the trial to be used to ensure that the trial is as efficient as possible 1.

Several authors have developed methods for such trials that test a single hypothesis to compare an experimental treatment with a control. These include the group sequential method 1, 2, the combination test method 3, 4, 5 and the conditional error function method 6, 7. Building on this work, there has been much recent interest in trials involving selection of the most promising of a number of treatments, in what is sometimes called a *multi‐arm, multi‐stage* (MAMS) design, an *adaptive seamless design*, or a *seamless phase II/III trial* 8, 9, or of a subgroup of the population in which a therapy is particularly effective 10, 11.

A desire in the analysis of trials that use interim analysis data for treatment selection is usually the control of the overall type I error rate, and this has been the focus of most of the development of statistical methodology in the area. In trials in which multiple hypotheses are tested, it is usually required that the familywise error rate is controlled in the strong sense. Several authors have developed methods based on the group sequential approach. Compared with other approaches, the group sequential method benefits from the fact that inference is based on sufficient statistics for the parameters of interest 12, 13, 14 and may be preferable from a regulatory perspective 15. The group sequential approach lacks flexibility, however, in that the rules for adaptation, in this case treatment selection, must generally be specified in advance in order to ensure type I error rate control. This has been termed *pre‐specified adaptivity*.

A number of authors 8, 16, 17, 18, 19, 20, 21, 22 have proposed group sequential methods for selection of one or more treatments using a pre‐specified rule. The selection rules specify either the conditions under which treatments should be dropped, as proposed by Follman 17, Hellmich 18 and Magirr *et al.* 22, or the number of treatments to continue at each stage, as proposed by Thall *et al.* 16, Stallard and Todd 8, Bischoff and Miller 19 and Stallard and Friede 20. Here, we take the latter approach and consider two‐stage designs in which the data available at the interim analysis are used for selection of a single experimental treatment, which continues along with the control to the second stage.

Thall *et al.* 16 and Stallard and Todd 8 show how the type I error rate can be controlled when selection of a single experimental treatment is based on the primary endpoint data alone and the most promising treatment is selected. Jennison and Turnbull 23 and Graf *et al.* 24 point out that the method proposed by Thall *et al.* 16 and Stallard and Todd 8 also controls the type I error rate when the treatment selected is not the most promising.

In some cases, in addition to primary endpoint data, data on some short‐term endpoint may also be used for treatment selection. As such data may be observed more quickly than the primary endpoint, short‐term endpoint data may be available at the interim analysis for patients for whom the primary endpoint data are not yet available. Stallard 21 showed how to control the type I error rate in this case, again assuming that the most promising experimental treatment was selected to continue beyond the interim analysis. The aim of this paper is to explore the properties of this approach when some treatment other than the most promising is selected, in particular when the selection rule is not specified in advance. We show that in this case, unlike the setting in which primary endpoint data only are used at the interim analysis considered by Jennison and Turnbull 23 and Graf *et al.* 24, such selection may lead to inflation of the type I error rate. Based on consideration of a trial in which the selection is made so as to maximise the conditional type I error given the data available at the interim analysis, we show how to construct critical values for the final hypothesis test to ensure that the familywise type I error rate is controlled strongly for any selection rule.

## 2 Motivating case study

There is a growing number of examples of multi‐arm clinical trials that incorporate treatment selection at an interim analysis, illustrating the range of areas of application and variety of details of implementation of treatment selection and interim analyses, with four examples given in the recent paper by Cuffe *et al.* 25.

Wilkinson and Murray 26 describe a multi‐stage phase II trial in Alzheimer's disease in which patients are initially randomised between a placebo control and 18, 24 and 36 mg/day doses of the exploratory drug, galantamine. In this study, the primary endpoint was a 3‐month change in Alzheimer's Disease Assessment Scale cognitive subsection (ADAS‐Cog) score, but both 3‐month and 6‐week change data were used at an interim analysis for dose selection. Wilkinson and Murray found the 24 mg/day dose to be the most promising, although further research 27 indicated that lower doses may be as efficacious.

In the setting of a phase II/III trial in Alzheimer's disease, the primary endpoint might be change in ADAS‐Cog score over 6 months, but, as in the trial described by Wilkinson and Murray, data on the change over a shorter period, for example, over 3 months, might be recorded and be available more rapidly than the final endpoint data. A decision at an interim analysis of which dose should continue along with the control to the second stage of the trial could thus be based on a combination of 6‐ and 3‐month data. Trials such as this motivate the work described in this paper. The aim is to provide a method that strongly controls the familywise type I error rate while allowing for use of all interim analysis data for treatment selection.

## 3 Error rate control for flexible selection of a single treatment in a two‐stage multi‐arm clinical trial

Consider a multi‐arm clinical trial comparing *k* ( k≥2) experimental treatments, treatments 1,…,*k*, with a control treatment, treatment 0. Let *θ*_*i*_ be a measure of the efficacy of treatment *i* relative to treatment 0 for *i* = 1,…,*k*, in terms of the primary endpoint and suppose that we wish to test a family of null hypotheses *H*_*i*_,*i*∈{1,…,*k*}, with *H*_*i*_:*θ*_*i*_≤*θ*_0_ for specified *θ*_0_, which, without loss of generality, we may take to be zero. We wish to control the familywise error rate in the strong sense for testing this family of null hypotheses.

Suppose that the trial is conducted in two stages, with, in the first stage, primary endpoint responses available for *n*_1_ patients randomised to each of treatments 0,…,*k*. Let *X*_1_ denote the full data observed in the first stage, which includes the primary responses from these *n*_1_ patients in each treatment group but, as in the example described earlier, may also include data from additional patients from whom primary endpoint data are not yet available.

On the basis of the data *X*_1_, treatment *T*(*X*_1_), for some *T*(*X*_1_)∈{1,…,*k*}, will be chosen to continue along with the control to the second stage with the hypothesis, $H_{T(X_{1})}$ tested at the end of the trial. Primary responses are then observed for a further *n*_2_−*n*_1_ patients randomised to each of treatments *T*(*X*_1_) and 0 in the second stage of the trial. Let *S*_*i*_ denote a test statistic for *H*_*i*_ based on data observed in both stages of the trial. We assume that the distribution of *S*_*i*_ depends on *θ*_*i*_, but not on $\theta_{i^{\prime }}$ with *i*′≠*i*, and that a larger *θ*_*i*_ value leads to larger values of *S*_*i*_, as formalised in the Appendix. The hypothesis $H_{T(X_{1})}$ will be rejected at the end of the trial if and only if $S_{T(X_{1})}\geq c$ for some critical value *c*, which will be chosen so as to provide strong familywise error rate control.

As indicated earlier, in general, the data *X*_1_ may include data other than the primary responses summarised by *S*_1_,…,*S*_*k*_ and so may depend on further parameters in addition to *θ*_1_,…,*θ*_*k*_. When this is the case, we will denote these additional parameters by $\theta_{k+1},\ldots ,\theta_{k^{*}}$ with *k* ^∗^>*k*. Otherwise, we will define *k* ^∗^=*k*. We will write ***θ*** for the vector of all parameters, that is, $\theta ={\left(\theta_{1},\ldots ,\theta_{k^{*}}\right)}^{\prime }$.

We require to strongly control the familywise error rate at level *α*, that is, to ensure that the probability of rejecting any true *H*_*i*_(*i* = 1,…,*k*) is at most *α* for any ***θ***. This is required for any data‐dependent choice of *T*(*X*_1_). We therefore wish to find *c* such that

(1) $${\mathrm{pr}}_{\theta }(S_{T(X_{1})}\geq c,\theta_{T(X_{1})}\leq 0)\leq \alpha$$

for all *θ*, and for any *T* in T, where T denotes the set of functions from the stage 1 sample space to {1,…,*k*}.

Given ***θ*** with *θ*_*i*_≤0, that is *H*_*i*_ true, for some *i* = 1,…,*k*, let

$$T_{\theta }^{*}(X_{1})={\arg\max}_{i\in \{1,\ldots ,k\}:\theta_{i}\leq 0}\left\{{\mathrm{pr}}_{\theta }(S_{i}\geq c|X_{1})\right\}.$$

Thus, $T_{\theta }^{*}$ denotes the rule that selects the treatment, treatment *i*, for which the conditional probability given *X*_1_ of rejecting *H*_*i*_ is highest amongst those *i* for which *H*_*i*_ is true given ***θ***. That is, $T_{\theta }^{*}(X_{1})$ is chosen to maximise the conditional error given *X*_1_ under ***θ***.

It can be shown (Appendix) that the left‐hand side of (1) is maximised over T∈T by taking $T(x_{1})=T_{\theta }^{*}(x_{1})$ for all *x*_1_ and maximised over *θ*_1_,…,*θ*_*k*_ by taking *θ*_1_=⋯=*θ*_*k*_=0.

To satisfy (1) and control the familywise error rate in the strong sense, it is therefore sufficient to have

(2) $$\mathrm{pr}\left(S_{T_{\theta }^{*}(X_{1})}\geq c;\theta_{1}=\cdots =\theta_{k}=0\right)\leq \alpha$$

for all *θ*_*k* + 1_,…,*θ* *k* ^*^.

In the case that *k* = *k* ^*^, when no short‐term endpoint data are available, (2) states that the familywise error rate is controlled at level *α* in the weak sense, that is, under the global null hypothesis ∩_*i* = 1,…,*k*_ *H*_*i*_. The result in the Appendix shows that strong control of the familywise type I error rate is also achieved. For *k* = *k* ^*^, ***θ*** and hence $T_{\theta }^{*}$ are entirely defined. In order to find the value of the critical value *c* to satisfy (2) and hence (1), it is thus necessary to obtain the distribution of $S_{T_{\theta }^{*}(X_{1})}$. Although in general this may not be a straightforward problem, the distribution is obtained for normally distributed responses by Thall *et al.* 16 and Stallard and Todd 8.

In general for *k* ^*^>*k*, the probability in (1) must be controlled for all $\theta_{k+1},\ldots ,\theta_{k^{*}}$. In this case, it is therefore necessary to find the values of $\theta_{k+1},\ldots ,\theta_{k^{*}}$ to maximise the probability in (2) to obtain the critical value *c*. Depending on the setting, this may be possible. For example, it may be that the values of $\theta_{k+1},\ldots ,\theta_{k^{*}}$ to maximise the error rate can be found directly, or that the probability in (2) does not depend on $\theta_{k+1},\ldots ,\theta_{k^{*}}$. The latter situation arises when data from different patients are independent and treatment selection is based either only on data from patients for whom primary endpoint data are available or on a combination of primary endpoint data and short‐term endpoint data available for patients for whom the primary endpoint is not yet available, as discussed in more detail in the next section.

## 4 Application with normally distributed responses and short‐term endpoint data

In this section, the method described earlier is illustrated through application in the setting of a clinical trial with a normally distributed primary endpoint. In many cases, similar settings with non‐normal data may be handled using asymptotically normally distributed test statistics as described in the group sequential setting by Jennison and Turnbull 28.

Suppose that in stage 1, primary endpoint data, *Y*_*ij*_, are observed for patients *j* = 1,…,*n*_1_ receiving treatment *i* and that in stage 2, data *Y*_*ij*_ are additionally observed for patients *j* = *n*_1_+1,…,*n*_2_ for the control, *i* = 0 and *i* = *T*(*X*_1_). Assume *Y*_*ij*_∼*N*(*μ*_*i*_,*σ* ^2^), with data from different patients independent, that is, $\mathrm{cov}(Y_{\mathrm{ij}},Y_{i^{\prime },j^{\prime }})=0$ unless *i* = *i*′ and *j* = *j*′, and let *θ*_*i*_=*μ*_*i*_−*μ*_0_(*i* = 1,…,*k*).

We assume that at the first stage, we additionally observe short‐term endpoint data, *W*_*ij*_, for patients *j* = 1,…,*N*_1_ in treatment group *i*, *i* = 0,…,*k*, where *N*_1_>*n*_1_, and that at the end of the second stage, data are available for $n_{2}\geq N_{1}$ patients per group including the *N*_1_ with short‐term endpoint data available at the first stage. We assume

(3) $$\left(\begin{array}{c} Y_{\mathrm{ij}}\\ W_{\mathrm{ij}} \end{array}\right)∼N\left(\left(\begin{array}{c} \mu_{i}\\ \mu_{k+1+i} \end{array}\right),\left(\begin{array}{cc} \sigma^{2} & \rho \sigma \sigma_{0}\\ \rho \sigma \sigma_{0} & \sigma_{0}^{2} \end{array}\right)\right),$$

with $\mathrm{cov}(W_{\mathrm{ij}},W_{i^{\prime }j^{\prime }})=0,\mathrm{cov}(Y_{\mathrm{ij}},Y_{i^{\prime }j^{\prime }})=0$, and $\mathrm{cov}(W_{\mathrm{ij}},Y_{i^{\prime }j^{\prime }})=0$(*i* ≠ *i*′ or *j* ≠ *j*′). Noting that *μ*_*k* + 1_ is the mean for the short‐term endpoint for the control treatment, the short‐term treatment effect for treatment *i* is given by *μ*_*k* + 1+*i*_−*μ*_*k* + 1_, which will be denoted *θ*_*k* + *i*_, (*i* = 1,…,*k*). The parameters of interest are *θ*_*i*_, *i* = 1,…,*k*, and it is desired to test the null hypotheses *H*_*i*_:*θ*_*i*_≤0(*i* = 1,…,*k*). The short‐term endpoint treatment effects, *θ*_*k* + *i*_(*i* = 1,…,*k*), are not of interest, so that we are interested in testing *k* null hypotheses, with the distribution of the data depending on *k* ^*^=2*k* parameters.

Assume that at the end of the trial a test statistic $S_{i}=\overset{n_{2}}{\underset{j=1}{\sum }}\left(Y_{\mathrm{ij}}-Y_{0j}\right)$ will be used for testing hypothesis *H*_*i*_. The results stated earlier and proved in the Appendix show that in order to find *c* to satisfy (1), we need to consider only the case *θ*_1_=⋯=*θ*_*k*_=0 and $T(x_{1})=T_{\theta }^{*}(x_{1})={\arg\max}_{i\in \{1,\ldots ,k\}:\theta_{i}\leq 0}\left\{{\mathrm{pr}}_{\theta }(S_{i}\geq c|X_{1}=x_{1})\right\}$. The resulting form of the rule, $T_{\theta }^{*}(x_{1})$, that maximises the conditional error over $\theta_{k+1},\ldots ,\theta_{k^{*}}$ is obtained later.

We consider first the case with *σ*, *σ*_0_ and *ρ*, assumed known. It can be shown that, given $X_{1}={\left(W_{1},\ldots ,W_{N_{1}},Y_{1},\ldots ,Y_{n_{1}}\right)}^{\prime }={\left(w_{1},\ldots ,w_{N_{1}},y_{1},\ldots ,y_{n_{1}}\right)}^{\prime }$, the conditional distribution of *S*_*i*_ is given by

$$S_{i}∼N\left((n_{2}-n_{1})\theta_{i}+n_{1}{\tilde{\theta }}_{i},2\left((n_{2}-n_{1})-(N_{1}-n_{1})\rho^{2}\right)\sigma^{2}\right),$$

where

$${\tilde{\theta }}_{i}={\overset{˘}{\theta }}_{i}+\rho \frac{\sigma }{\sigma_{0}}\overset{N_{1}}{\underset{j=n_{1}+1}{\sum }}(w_{\mathrm{ij}}-w_{0j}-\theta_{k+i}))/n_{1}$$

with ${\overset{˘}{\theta }}_{i}=\overset{n_{1}}{\underset{j=1}{\sum }}(y_{\mathrm{ij}}-y_{0j})/n_{1}$ denoting the estimate of *θ*_*i*_ based on the interim primary endpoint data alone.

Thus, if *θ*_1_=⋯=*θ*_*k*_=0, $T_{\theta }^{*}(x_{1})=\arg\underset{i\in \{1,\ldots ,k\}}{\max}{\tilde{\theta }}_{i}$. The selection rule $T_{\theta }^{*}(X_{1})$ would maximise the error rate if $\theta_{k+1},\ldots ,\theta_{k^{*}}$ were known. Even if $\theta_{k+1},\ldots ,\theta_{k^{*}}$ are unknown, however, full flexibility over the choice of *T* means that this rule might be chosen. The critical value, *c*, must thus be calculated based on this rule to ensure error rate control.

The joint distribution of ${\tilde{\theta }}_{1},\ldots ,{\tilde{\theta }}_{k}$ and *S*_1_,…,*S*_*k*_ is given by

$${\left({\tilde{\theta }}_{1},\ldots ,{\tilde{\theta }}_{k},S_{1},\ldots ,S_{k}\right)}^{\prime }∼N\left({\left(\theta_{1},\ldots ,\theta_{k},\theta_{1},\ldots ,\theta_{k}\right)}^{\prime },\left(\begin{array}{cc} V_{1} & \rho_{e}\sqrt{V_{1}V_{2}}\\ \rho_{e}\sqrt{V_{1}V_{2}} & V_{2} \end{array}\right)⊗\Sigma \right)$$

with $V_{1}=2\sigma^{2}(n_{1}+\rho^{2}(N_{1}-n_{1}))/n_{1}^{2}$, *V*_2_=2*n*_2_ *σ* ^2^ and $\rho_{e}=\sqrt{(n_{1}+\rho^{2}(N_{1}-n_{1}))/n_{2}}$. As this distribution does not depend on $\theta_{k+1},\ldots ,\theta_{k^{*}}$, the distribution of $S_{T_{\theta }^{*}(X_{1})}$ does not depend on $\theta_{k+1},\ldots ,\theta_{k^{*}}$ and is entirely specified by taking *θ*_1_=⋯=*θ*_*k*_=0.

Noting that the conditional variance of *S*_*i*_ given ${\tilde{\theta }}_{i}$ is $V_{2}\left(1-\rho_{e}^{2}\right)$, $S_{T_{\theta }^{*}(X_{1})}$ has density

$$\overset{k}{\underset{i=1}{\sum }}\overset{\infty }{\underset{-\infty }{\int }}\frac{1}{\sqrt{V_{2}\left(1-\rho_{e}^{2}\right)}}\varphi \left(\frac{s-(n_{2}-n_{1})\theta_{i}-n_{1}x}{\sqrt{V_{2}\left(1-\rho_{e}^{2}\right)}}\right)f_{i}(x,V_{1})\mathrm{dx}$$

anddistribution function

$$\overset{k}{\underset{i=1}{\sum }}\overset{\infty }{\underset{-\infty }{\int }}\Phi \left(\frac{s-(n_{2}-n_{1})\theta_{i}-n_{1}x}{\sqrt{V_{2}\left(1-\rho_{e}^{2}\right)}}\right)f_{i}(x,V_{1})\mathrm{dx},$$

where

$$f_{i}(x,V)=\overset{\infty }{\underset{-\infty }{\int }}\frac{1}{V}\varphi \left(\frac{x-y}{\sqrt{V}}\right)\varphi \left(\frac{y-\theta_{i}}{\sqrt{V}}\right)\underset{i^{\prime }\neq i}{\prod }\Phi \left(\frac{y-\theta_{i}}{\sqrt{V}}\right)\mathrm{dy}$$

and *φ* and Φ denote the density and distribution functions of the standard normal distribution 8. Numerical integration and a simple numerical search, for example, using the R function uniroot, can thus be used to find the critical value *c* to control the error rate as required.

When *σ*, *σ*_0_ and *ρ* are not known, estimates obtained at the interim analysis can be used in this expression to find *c*.

## 5 Example

In this section, the method described earlier is illustrated through application to a simulated dataset based on the setting of the phase II/III trial described in Section 2. We assume that three doses, 16, 24 and 32 mg/day of galantamine, are compared with a placebo control. In the first stage of the trial, *N*_1_=100 patients per group are randomised between the four treatment arms, with an interim analysis being conducted when *w*_*ij*_, the 3‐month endpoint data, are available for all of these patients. It is assumed that at this time *y*_*ij*_, the primary, 6 month, endpoint data, are available for *n*_1_=40 patients per arm. Data were simulated from a model based on the Cochrane review of clinical trials of galantamine by Loy and Schneider 27.

**Figure 1:**
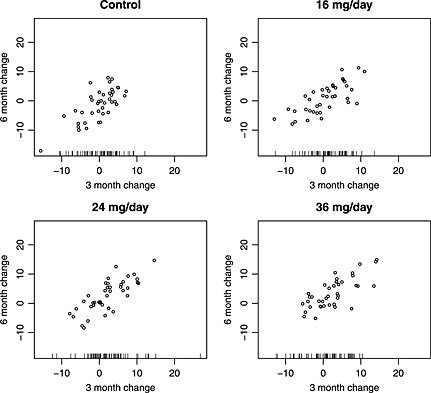
Interim analysis data for simulated example (see main text for details).

The simulated interim analysis data are shown in Figure 1 giving the change from baseline to 3‐ and 6‐month ADAS‐Cog scores with the sign of the change chosen so that positive changes correspond to an improvement. Plotted points indicate values of the long‐term and short‐term endpoint data for the *n*_1_ patients per group for whom these are both available. The tick marks at the bottom of each plot give the values of the short‐term endpoint data for the remaining *N*_1_−*n*_1_ patients per group for whom long‐term endpoint data are not available at this time. The mean long‐term response for the *n*_1_ patients in each group are given in Table 1 along with the mean short‐term response for these *n*_1_ patients per group, for the additional *N*_1_−*n*_1_ patients per group for whom only short‐term endpoint data are available and for all *N*_1_ patients per group included in the interim analysis.

**Table 1:** Summary of interim analysis results for simulated example.

Dose	$\frac{\overset{n_{1}}{\underset{j=1}{\sum }}y_{\mathrm{ij}}}{n_{1}}$	$\frac{\overset{n_{1}}{\underset{j=1}{\sum }}w_{\mathrm{ij}}}{n_{1}}$	$\frac{\overset{N_{1}}{\underset{j=n_{1}+1}{\sum }}w_{\mathrm{ij}}}{N_{1}-n_{1}}$	$\frac{\overset{N_{1}}{\underset{j=1}{\sum }}w_{\mathrm{ij}}}{N_{1}}$	${\overset{˘}{\theta }}_{i}$	${\hat{\theta }}_{i}$	${\tilde{\theta }}_{k}$
Control (*i* = 0)	−1.24	−0.05	0.06	0.02			
16 mg/day (*i* = 1)	0.91	0.56	1.48	1.11	2.15	2.53	2.06
24 mg/day (*i* = 2)	2.64	2.15	2.43	2.32	3.88	3.96	3.74
32 mg/day (*i* = 3)	3.62	2.59	0.36	1.25	4.86	3.77	2.30

Using the approach of Stallard and Todd 8, treatment selection would be based on the long‐term endpoint data alone. The estimate of the treatment effect for treatment *i* relative to the control, *θ*_*i*_, is then given by ${\overset{˘}{\theta }}_{i}=\overset{n_{1}}{\underset{j=1}{\sum }}(y_{\mathrm{ij}}-y_{0j})/n_{1}$, which is given in Table 1. In this case, this would lead to selection of the 32 mg/kg dose. Assuming treatment selection is always made using this approach, the critical value for the final test statistic needs to be adjusted as described by Stallard and Todd 8 in order to control the type I error rate. In this case, the critical value depends on *n*_1_ and *N*. The critical value, $c/\sqrt{V_{2}}$, with which a standardised test statistic should be compared with give a one‐sided type I error rate of *α* = 0.025 is 2.19.

Use of the additional 3‐month endpoint data would improve the treatment selection. The method of Stallard 21 would base treatment selection on the estimate of *θ*_*i*_ given by

$${\hat{\theta }}_{i}={\overset{˘}{\theta }}_{i}-\rho \frac{\sigma }{\sigma_{0}}\overset{n_{1}}{\underset{j=1}{\sum }}\left(w_{\mathrm{ij}}-w_{0j}-{\hat{\theta }}_{k+i}\right)/n_{1},$$

with ${\hat{\theta }}_{k+i}=\overset{N_{1}}{\underset{j=1}{\sum }}(w_{\mathrm{ij}}-w_{0j})/N_{1}$. These estimates are also given in Table 1. Figure 1 shows that for *i* = 3 values of *w*_*ij*_ for *j* = *n*_1_,…,*N*_1_ are typically smaller than those for *j* = 1,…,*n*_1_, as is reflected in the mean values given in Table 1. This leads to ${\hat{\theta }}_{3}$ being considerably smaller than ${\overset{˘}{\theta }}_{3}$, so that ${\hat{\theta }}_{3}<{\hat{\theta }}_{2}$. Incorporation of the additional short data would thus lead to selection of the 24 mg/day dose rather than 32 mg/day. If this approach is always used for treatment selection, the critical value needs to be adjusted to allow for the use of the additional 3‐month endpoint information as described by Stallard 21. In this case, the critical value depends on *n*_1_, *N*_1_, *N* and the correlation between the endpoints, *ρ*. Using an estimate of *ρ* from the interim analysis data, in this case, 0.77, leads to a critical value with which a standardised test statistic should be compared of 2.23.

As described earlier, the conditional type I error rate is maximised by selecting the treatment based on the estimate ${\tilde{\theta }}_{i}$. This requires specification of the true short‐term endpoint treatment effects *θ*_*k* + *i*_,*i* = 1,…,3. Based on 27, for example, a researcher might guess that the true treatment effects relative to the control might be close to 1.5, 2.5 and 2.5 for *i* = 1,2 and 3, that is, for the 16, 24 and 32 mg/day doses. The values of ${\tilde{\theta }}_{k}$ obtained using these values for *k* + *i* are given in Table 1. In this case, this would again lead to selection of the 24 mg/day dose. If a larger value was assumed for *θ*_*k* + *i*_, ${\tilde{\theta }}_{i}$ would be reduced, reflecting the fact that the observed values of *w*_*ij*_ are smaller relative to their expected value. Thus, for example, if it was assumed that the true treatment effects for the three doses were 1.5, 4.0 and 4.0, ${\tilde{\theta }}_{2}$ and ${\tilde{\theta }}_{3}$ would be smaller than ${\tilde{\theta }}_{1}$, so that the 16 mg/day dose would be selected. Note that using the estimated values for *θ*_*k* + *i*_, *i* = 1,…,3 based on the interim analysis data, denoted by ${\hat{\theta }}_{k+i}$ earlier, leads to ${\tilde{\theta }}_{i}$ equal to ${\hat{\theta }}_{i}$ given earlier, reflecting the fact that no additional information is available in this case.

As this selection maximises the type I error given the observed data, in the case of fully flexible treatment selection, selection based on ${\tilde{\theta }}_{i}$ could lead to inflation of the type I error rate for appropriate choice of *θ*_*k* + *i*_, *i* = 1,…,3 unless the critical value was adjusted to allow for this. In this case, the critical value that controls the type I error rate again depends on *n*_1_, *N*_1_, *N* and *ρ*. Using the estimated value of *ρ* as mentioned earlier, the critical value for a standardised test statistic is 2.25.

Assuming selection of the 24 mg/day dose to continue to the second stage, simulated means at the end of the trial for the 6‐month change for control and 24 mg/day arms were −1.39 and 2.07, respectively, with an estimated standard error for the difference between them, $\sqrt{V_{2}}$, of 0.525, leading to standardised test statistic of 6.60. A significant treatment effect is thus indicated for this dose irrespective of the way in which it was selected.

## 6 Simulation study

In order to illustrate the properties of the method described and to compare them with those of alternative approaches, it is of interest to consider outcomes from a large number of simulated trials under specified scenarios.

One alternative to the phase II/III trial considered earlier would be to conduct separate phase II and phase III trials, the first used for selection of the most promising dose and the second comparing this dose with a placebo. As previous simulations (for example, 29) have shown that such an approach can require considerably more patients than a two‐stage phase II/III design, this option was not included in our simulation study.

We considered two‐stage trials with, as mentioned earlier, *α* = 0.025,*k* = 3,*n*_1_=40,*N*_1_=100 and *n*_2_=200. Critical values with which standardised test statistics, $S_{i}/\sqrt{V_{2}}$, would be compared in this trial using the earlier approach to control the type I error rate for any treatment selection rule are given in Table 2 for a range of *ρ* values calculated assuming *σ*,*σ*_0_ and *ρ* are known. The critical values increase with *ρ* to control the type I error rate. Results from simulations of this procedure are shown in columns 3 to 5 of the table. Estimated error rates were based on 100 000 simulations so that if the true type I error rate was 0.025, we would expect the estimated error rate to be below 0.026 with probability 0.975. Columns 3 and 4 show the type I error rates simulated under *θ*_1_=⋯=*θ*_*k*_=0 with selection of $\arg\max\{{\tilde{\theta }}_{i}\}$ assuming $\theta_{k+1},\ldots ,\theta_{k^{*}}$ known, when the type I error rate is maximised, both using the known values of *σ*, *σ*_0_ and *ρ* and using estimates of these obtained from the stage 1, respectively, confirming that the type I error rate is controlled at the nominal 0.025 level in both cases.

**Table 2:** Properties of the flexible design with *α* = 0.025,*k* = 3,*n*_1_=40, *N*_1_=100,*n*_2_=200 and *σ* = *σ*_0_=1 for a range of *ρ* values (error rates are based on 100 000 simulations).

*ρ*	$c/\sqrt{V_{2}}$	Type I error selecting $\arg\max\{{\tilde{\theta }}_{i}\}$	Type I error selecting $\arg\max\{{\tilde{\theta }}_{i}\}$		Type I error selecting $\arg\max\{{\hat{\theta }}_{i}\}$	Power selecting $\arg\max\{{\hat{\theta }}_{i}\}$
		Variances known	Variances unknown		Variances known	Variances unknown
0.0	2.19	0.0242	0.0246		0.0243	0.7827
0.5	2.22	0.0241	0.0244		0.0234	0.7974
0.6	2.23	0.0245	0.0248		0.0233	0.8071
0.7	2.24	0.0249	0.0252		0.0239	0.8119
0.8	2.25	0.0253	0.0255		0.0240	0.8251
0.9	2.27	0.0245	0.0248		0.0236	0.8358

In practice, as $\theta_{k+1},\ldots ,\theta_{k^{*}}$ are unknown, selection based on ${\tilde{\theta }}_{i}$ is impossible. An alternative would be to select the treatment with the largest estimated effect ${\hat{\theta }}_{i}$ as described in the earlier example, with ${\hat{\theta }}_{k+i}=\overset{N_{1}}{\underset{j=1}{\sum }}(w_{\mathrm{ij}}-w_{0j})/N_{1}$ as in Stallard 21. Column 5 shows the type I error rate in this case, when the test is conservative.

The last column in Table 2 shows the power to select treatment 1 and reject *H*_1_ when *θ*_1_=1/3,*θ*_2_=*θ*_3_=0 again selecting $\arg\overset{k}{\underset{i=1}{\max}}\{{\hat{\theta }}_{i}\}$ with $\theta_{k+1},\ldots ,\theta_{k^{*}}$ unknown. The power shows how the gain from using the short‐term endpoint increases with *ρ* relative to using the long‐term endpoint alone, equivalent to *ρ* = 0.

For comparison with Table 2, Table 3 gives critical values, $c/\sqrt{V_{2}}$, from the method proposed by Stallard 21, assuming the selection of $\arg\overset{k}{\underset{i=1}{\max}}\{{\hat{\theta }}_{i}\}$, and Stallard and Todd 8, assuming the selection of $\arg\overset{k}{\underset{i=1}{\max}}\{{\overset{˘}{\theta }}_{i}\}$, and the resulting simulated type I error rate under *θ*_1_=⋯=*θ*_*k*_=0 with the treatment selected so as to maximise the error rate, that is, selecting $\arg\max\{{\tilde{\theta }}_{i}\}$, if $\theta_{k+1},\ldots ,\theta_{k^{*}}$ were known, in this case using the known values of *σ*, *σ*_0_ and *ρ*. The error rate inflation from use of the Stallard and Todd 8 method when selecting $\arg\overset{k}{\underset{i=1}{\max}}\{{\hat{\theta }}_{i}\}$ was demonstrated by Stallard 21. The new results show that the error rate may be inflated further by using some other selection rule and illustrate the maximal error rate inflation from the use of any selection rule based on *X*_1_ for these two designs.

**Table 3:** Properties of the Stallard 21 and Stallard and Todd 8 designs and the combination test with parameters as in Table 1 (error rates are based on 100 000 simulations).

*ρ*	Stallard test			Stallard and Todd test			Combination test	
	$c/\sqrt{V_{2}}$	Type I error		$c/\sqrt{V_{2}}$	Type I error		Type I error	Power
		selecting			selecting		selecting	selecting
		$\arg\max\{{\tilde{\theta }}_{i}\}$			$\arg\max\{{\tilde{\theta }}_{i}\}$		$\arg\max\{{\tilde{\theta }}_{i}\}$	$\arg\max\{{\hat{\theta }}_{i}\}$
0.0	2.19	0.0242		2.19	0.0242		0.0197	0.7617
0.5	2.20	0.0252		2.19	0.0259		0.0200	0.7807
0.6	2.21	0.0256		2.19	0.0270		0.0200	0.7912
0.7	2.22	0.0262		2.19	0.0284		0.0216	0.8010
0.8	2.23	0.0266		2.19	0.0296		0.0218	0.8134
0.9	2.25	0.0252		2.19	0.0298		0.0231	0.8265

Additional simulations were conducted with *n*_1_=20,*N*_1_=50 and *n*_2_=100 and *n*_1_=10,*N*_1_=25 and *n*_2_=50 to explore the impact of smaller sample sizes when *σ*, *σ*_0_ and *ρ* were considered unknown. There was some indication of type I error rate inflation in these cases with maximum simulated error rates for the new method of 0.02601 and 0.02645, respectively. This suggests that the new method may not be suitable in trials of very rare diseases or orphan drugs when sample sizes as small as this may be used. In other settings, it is unlikely that such small sample sizes would be used for a confirmatory clinical trial.

An alternative method to that proposed here would be to use a combination testing method 3, 9, 30, as this is known to strongly control the type I error rate for any treatment selection. To apply such an approach in this case requires some care to ensure that the *p*‐values that are combined satisfy the ‘p‐clud’ condition 31. Friede *et al.* 32 describe one way in which this can be performed, considering the ‘stage 1’ *p*‐value to be that obtained from the analysis of the primary endpoint of all those *N*_1_ patients per group for whom some data were available at the interim analysis, and the ‘stage 2’ *p*‐value to be from the analysis of the primary endpoint for the *n*_2_−*N*_1_ patients per group recruited following the treatment selection. The power for this method to select treatment 1 and reject *H*_1_ when *θ*_1_=1/3,*θ*_2_=*θ*_3_=0 again selecting $\arg\overset{k}{\underset{i=1}{\max}}\{{\hat{\theta }}_{i}\}$ and assuming *σ*,*σ*_0_ and *ρ* are known is also shown in Table 3 for comparison with that of the new procedure shown in Table 2. It can be seen that the combination test has slightly lower power in this case. This is consistent with the findings of Friede and Stallard 33. Although the power gain for the new procedure over the combination test is modest, in settings in which it is known that a single experimental treatment will be selected to continue along with the control to the second stage, the new method is to be preferred. If additional flexibility, for example, over the number of treatments to continue, is required, the combination test could be used with only a small loss in power.

## 7 Discussion

This paper has considered trials in which treatment selection at an interim analysis may be made using data for patients for whom the primary endpoint has not yet been observed. Stallard 21 showed how the error rate can be controlled in this case if the most promising treatment is selected. We have shown that this does not provide error rate control for an arbitrary selection rule and illustrated how this can be achieved.

It is interesting to consider possible extensions of the method proposed. One extension is to allow the possibility of early stopping at the interim analysis with additional testing of *H*_*i*_(*i* = 1,…,*k*) on the basis of *X*_1_. In this case, *H*_*i*_ could be tested using some test statistic $S_{i}^{\left(1\right)}(X_{1})$, rejecting *H*_*i*_ if and only if $S_{i}^{\left(1\right)}(X_{1})$ is at least as large as some specified value *c*_1_. If any *H*_*i*_ is rejected, that hypothesis will not be tested again, so that treatment *i* could be dropped from the trial, and if not all hypotheses are rejected, $H_{T(x_{1})}$ is selected and tested at the end of the second stage, being rejected if and only if $S_{T(x_{1})}(X_{2})\geq c$. In this case, as there is an opportunity to reject hypotheses at both stages, requirement (1) is insufficient to give both *c*_1_ and *c* uniquely. A stronger requirement is that the probability to reject any true *H*_*i*_(*i* = 1,…,*k*) at or before analysis *j* is controlled to be at most some specified *α*_*j*_(*j* = 1,2) with *α*_1_≤*α*_2_=*α*. The values *α*_1_ and *α*_2_ may be considered as a simple *α*‐spending function as proposed by Slud and Wei 34.

A further extension would be to allow additional interim analyses, so that the two‐stage design becomes a multi‐stage design. With a single hypothesis selected at the first interim analysis, as no further selection is possible, the extension to allow additional stages with the opportunity for early stopping, for example, using an *α*‐spending function approach, is relatively straightforward. The extension of the method proposed to select more than one treatment to continue, and hence more than one hypothesis to be tested at the end of the trial, or in a multi‐stage trial to allow selection of treatments over several stages, while maintaining strong control of the familywise error rate, for example, to add flexibility to the methods of Follman *et al.* 17, Hellmich 18 or Magirr *et al.* 22, is more difficult. If *c* is fixed as above, with full flexibility over the choice of hypotheses from the family *H*_*i*_(*i* = 1,…,*k*), the type I error rate is maximised by selecting all *k* hypotheses, so that *c* must be chosen to control the type I error in this case. This can be achieved using a test similar to the Dunnett test. An alternative is to have *c* dependent on the number of hypotheses selected, say *k*_1_≤*k*. One such approach, using the conditional error method, has been proposed by Magirr *et al.* 35. If *k*_1_ is specified in advance, the method described can be extended for *k*_1_>1. In this case, a result analogous to Theorem 1 in the Appendix, stating that the type I error rate is maximised by selecting the *k*_1_ treatments corresponding to values of *i* with the largest values of ${\mathrm{pr}}_{\theta }(S_{i}\geq c|X_{1})$, can be shown to hold.

In this paper, we have assumed that the short‐term endpoint information available is based on the same length of follow‐up for all patients. An anonymous referee has suggested that short‐term information based on different follow‐up duration for different patients might be available. This would also be an interesting area for future research.

In this paper, we have considered flexible treatment selection. Other adaptations could, in principle, be handled in a similar way. Graf and Bauer 36 and Graf *et al.* 24, for example, considered sample size reestimation based on interim data in two‐stage studies both with and without treatment selection.

The method introduced in this paper enables strong control of the familywise error rate when the treatment selection rule is unspecified by constructing the selection rule that maximises the conditional error rate and then obtaining a critical value such that strong error rate control is achieved if this rule is used.

When treatment selection is made on the basis of the primary endpoint data available at the interim analysis, that is, in the setting considered by Thall *et al.* 16 and Stallard and Todd 8, this maximum occurs for selection of the most promising experimental treatment as noted by Jennison and Turnbull 23 and Graf *et al.* 24, so that the method of Thall *et al.* 16 and Stallard and Todd 8 strongly controls the familywise error rate if any other treatment is selected.

In addition, if the data available at the interim analysis, *X*_1_, include additional data available from the same patients from whom primary endpoint data are available, given the final endpoint data, *S*_*i*_ is conditionally independent of these additional data. The distribution of *S*_*i*_∣*X*_1_ thus depends on *θ*_*i*_ alone, and the probability in (2) does not depend on $\theta_{k+1},\ldots ,\theta_{k^{*}}$, so that strong control of the familywise error rate is again obtained. Similarly, the distribution of *S*_*i*_ given *X*_1_ can also reasonably be assumed to be independent of any data obtained from sources external to the trial, so that this can also be used for decision‐making without inflating the type I error rate. The latter point allows, for example, treatment selection using Bayesian methods where prior distributions may be informed, either formally or informally, by results from other trials of the same or similar treatments.

Although we have considered group sequential approaches, with *c* fixed, as indicated earlier, alternative methods with *c* depending on *X*_1_ can also be used to allow flexibility with strong control of the familywise error rate. Both the combination testing approach and the conditional error approach have been proposed for use in the treatment selection setting. Combination tests generally allow greater flexibility than the group sequential approach, in this case allowing fully flexible selection of any number of hypotheses for testing at the second stage, with some modification 32 even in the case of correlated data. As demonstrated in the earlier example, however, this can be at the loss of efficiency. Koenig *et al.* 37, Posch *et al.* 38 and Magirr *et al.* 35 have proposed methods based on the conditional error approach of Müller and Schäfer 7. Friede *et al.* 33 showed that the method of Koenig *et al.* 37 performed well in terms of power. Extending such methods to more general settings, such as multi‐stage designs or correlated data at different stages, could be difficult, however.

## Appendix 1

### 1.1

In a study in two stages, let *X*_1_ denote the first stage data with the distribution of *X*_1_ depending on $\theta ={\left(\theta_{1},\ldots ,\theta_{k^{*}}\right)}^{\prime }$. We wish to test a family of null hypotheses *H*_*i*_,*i*∈{1,…,*k*}, for some *k*≤*k* ^*^ with *H*_*i*_:*θ*_*i*_≤0, controlling the familywise error rate in the strong sense.

The study will be conducted so as to test a hypothesis, $H_{T(x_{1})}(T(x_{1})\in \{1,\ldots ,k\})$, chosen on the basis of *X*_1_. Further data will then be observed, leading to a final dataset *X*_2_, and $H_{T(x_{1})}$ will be rejected if and only if $S_{T(x_{1})}(X_{2})\geq c$ for some specified *c* and test statistic $S_{T(x_{1})}(X_{2})$. Writing *S*_*i*_ for *S*_*i*_(*X*_2_), we assume that the distribution of *S*_*i*_ depends on *θ*_*i*_, but on no other elements of *θ* and that for any possible stage 1 dataset, *x*_1_, *S*_*i*_ and *S*_*i*_∣*X*_1_=*x*_1_ is stochastically non‐decreasing in *θ*_*i*_.

Let $T_{\theta }^{*}(X_{1})={\arg\max}_{i\in \{1,\ldots ,k\}:\theta_{i}\leq 0}\left\{{\mathrm{pr}}_{\theta }(S_{i}\geq c|X_{1})\right\}$.

We prove the following result. Theorem 1

- For given *θ*, the left‐hand side of (1) is maximised over T∈T by taking $T(x_{1})=T_{\theta }^{*}(x_{1})$ for all *x*_1_.
- The left‐hand side of (1) is maximised over *θ*_1_,…,*θ*_*k*_ by taking *θ*_*i*_=0(*i* = 1,…,*k*).

The proof of Theorem 1 is based on the following two lemmas.
Lemma 1 Let T(t) be the set of functions from the sample space of *X*_1_ to {1,…,*t*}, for *t*≤*k*, and then for fixed *θ*, the left‐hand side of (1) is maximised over T∈T(t) by $T=T_{\theta }^{*(t)}$, where

$$T_{\theta }^{*(t)}(x_{1})={\arg\max}_{i\in \{1,\ldots ,t\}:\theta_{i}\leq 0}\left\{{\mathrm{pr}}_{\theta }(S_{i}\geq c|X_{1}=x_{1})\right\}.$$

The probability ${\mathrm{pr}}_{\theta }(S_{T(X_{1})}\geq c,\theta_{T(X_{1})}\leq 0)$ is equal to $E_{\theta }({\mathrm{pr}}_{\theta }(S_{T(X_{1})}\geq c,\theta_{T(X_{1})}\leq 0|X_{1}))$. This is maximised by taking T∈T(t) to maximise ${\mathrm{pr}}_{\theta }(S_{T(X_{1})}\geq c,\theta_{T(X_{1})}\leq 0|X_{1})$, that is by taking $T=T_{\theta }^{*(t)}$ as stated. □
Lemma 2 For fixed $\theta_{k+1},\ldots ,\theta_{k^{*}}$, the left‐hand side of (1) is non‐decreasing in *θ*_*i*_,*i* = 1,…,*k*.
As $T_{\theta }^{*}$ takes values in {*i*:*θ*_*i*_≤0}, we have ${\mathrm{pr}}_{\theta }\left(S_{T_{\theta }^{*}(X_{1})}\geq c,\theta_{T_{\theta }^{*}(X_{1})}\leq 0\right)={\mathrm{pr}}_{\theta }\left(S_{T_{\theta }^{*}(X_{1})}\geq c\right)$. This is equal to $E_{\theta }\left({\mathrm{pr}}_{\theta }\left(S_{T_{\theta }^{*}(X_{1})}\geq c|X_{1}\right)\right)$, which, by Lemma 1, is equal to $E_{\theta }\left(\underset{i:\theta_{i}\leq 0}{\max}\left\{{\mathrm{pr}}_{\theta }(S_{i}\geq c|X_{1})\right\}\right)$. It has been assumed that for any *x*_1_ and *i*, *S*_*i*_∣*X*_1_=*x*_1_ is stochastically non‐decreasing in *θ*_*i*_. Therefore, $\underset{i:\theta_{i}\leq 0}{\max}\left\{{\mathrm{pr}}_{\theta }\left(S_{i}\geq c|X_{1}=x_{1}\right)\right\}$ is non‐decreasing in *θ*_*i*_(*i* = 1,…,*k*). As this holds for all *x*_1_, the expected value must also be non‐decreasing in *θ*_*i*_ as required. □
Proof of Theorem 1
(1) Setting *t* = *k*, the first part of Theorem 1 follows from Lemma 1. For the second part, writing $\theta^{\left(t\right)}={\left(0,\ldots ,0,\theta_{t+1},\ldots ,\theta_{k^{*}}\right)}^{\prime }$, where the first *t* elements are equal to 0, we require

(1) $${\mathrm{pr}}_{\theta }\left(S_{T_{\theta }^{*}(X_{1})}\geq c,\theta_{T_{\theta }^{*}(X_{1})}\leq 0\right)\leq {\mathrm{pr}}_{\theta^{\left(k\right)}}\left(S_{T_{\theta^{\left(k\right)}}^{*}(X_{1})}\geq c,\theta_{T_{\theta^{\left(k\right)}}^{*}(X_{1})}\leq 0\right).$$

If *θ*_*i*_>0(*i* = 1,…,*k*), the left‐hand side of (1) is equal to zero, so that the inequality is trivially satisfied. Suppose, therefore, that *θ*_*i*_<0 for some *i*≤*k* and, without loss of generality, reorder such that *θ*_*i*_≤0 for *i* = 1,…,*t* and *θ*_*i*_>0 for *i* = *t* + 1,…,*k* for some *t*. As, by Lemma 2, ${\mathrm{pr}}_{\theta }(S_{T_{\theta }^{*}(X_{1})}\geq c,\theta_{T_{\theta }^{*}(X_{1})}\leq 0)$ is non‐decreasing in *θ*_*i*_, the probability on the left‐hand side of (1) must be non‐decreased by taking *θ*_1_=0(*i* = 1,…,*t*). Thus,

(2) $${\mathrm{pr}}_{\theta }\left(S_{T_{\theta }^{*}(X_{1})}\geq c,\theta_{T_{\theta }^{*}(X_{1})}\leq 0\right)\leq {\mathrm{pr}}_{\theta^{\left(t\right)}}\left(S_{T_{\theta^{\left(t\right)}}^{*}(X_{1})}\geq c,\theta_{T_{\theta^{\left(t\right)}}^{*}(X_{1})}\leq 0\right).$$

As $T_{\theta^{\left(t\right)}}^{*}$ maximises the left‐hand side of (1) under *θ* ^(*t*)^, we must have $T_{\theta^{\left(t\right)}}^{*}(X_{1})\in \{1,\ldots ,t\}$ because if, for any *x*_1_, it took a value outside this range, the probability would be increased by moving mass into this set. Thus, $T_{\theta^{\left(t\right)}}^{*}=T_{\theta^{\left(t\right)}}^{*(t)}$ and the right‐hand side of (2) is

$${\mathrm{pr}}_{\theta^{\left(t\right)}}\left(S_{T_{\theta^{\left(t\right)}}^{*(t)}(X_{1})}\geq c,\theta_{T_{\theta^{\left(t\right)}}^{*(t)}(X_{1})}\leq 0\right).$$

The function $T_{\theta^{\left(t\right)}}^{*(t)}$, and hence $T_{\theta^{\left(t\right)}}^{*}$, as defined by Lemma 1, depends only on *S*_1_,…,*S*_*t*_, and by Assumption 1 therefore not on *θ*_*t* + 1_,…,*θ*_*k*_. Thus, the right‐hand side of (2) is unchanged by setting *θ*_*i*_=0(*i* = *t* + 1,…,*k*) and is, as required, equal to

$$\begin{array}{c} {\mathrm{pr}}_{\theta^{\left(k\right)}}\left(S_{T_{\theta^{\left(k\right)}}^{*}(X_{1})}\geq c,\theta_{T_{\theta^{\left(k\right)}}^{*}(X_{1})}\leq 0\right). \end{array}$$

□
